# A Coarse Alignment Method Based on Digital Filters and Reconstructed Observation Vectors

**DOI:** 10.3390/s17040709

**Published:** 2017-03-29

**Authors:** Xiang Xu, Xiaosu Xu, Tao Zhang, Yao Li, Zhicheng Wang

**Affiliations:** 1 Key Laboratory of Micro-Inertial Instrument and Advanced Navigation Technology, Ministry of Education, Nanjing 210096, China; xuxiang@seu.edu.com (X.X.); zhangtao22@seu.edu.cn (T.Z.); liyao@seu.edu.cn (Y.L.); 2 School of Instrument Science and Engineering, Southeast University, Nanjing 210096, China; 3 School of Information and Control, Nanjing University of Information Science and Technology, Nanjing 210044, China; xxwyy@seu.edu.cn

**Keywords:** SINS, coarse alignment, digital filter, vector reconstruction, robust Kalman filter

## Abstract

In this paper, a coarse alignment method based on apparent gravitational motion is proposed. Due to the interference of the complex situations, the true observation vectors, which are calculated by the apparent gravity, are contaminated. The sources of the interference are analyzed in detail, and then a low-pass digital filter is designed in this paper for eliminating the high-frequency noise of the measurement observation vectors. To extract the effective observation vectors from the inertial sensors’ outputs, a parameter recognition and vector reconstruction method are designed, where an adaptive Kalman filter is employed to estimate the unknown parameters. Furthermore, a robust filter, which is based on Huber’s M-estimation theory, is developed for addressing the outliers of the measurement observation vectors due to the maneuver of the vehicle. A comprehensive experiment, which contains a simulation test and physical test, is designed to verify the performance of the proposed method, and the results show that the proposed method is equivalent to the popular apparent velocity method in swaying mode, but it is superior to the current methods while in moving mode when the strapdown inertial navigation system (SINS) is under entirely self-contained conditions.

## 1. Introduction

Initial alignment procedure is of vital importance in the strapdown inertial navigation system (SINS); the precision of the initial alignment will determine the positioning precision of SINS, and the poor initial alignment accuracy will end up with poor navigation. Thus, the higher precision of the initial alignment is fundamental to the more stable inertial positioning [1,2,3]. In order to acquire the high performance of initial alignment, many researchers are devoted to explore novel methods, which are focused on improving the alignment precision and the convergence rate, and a series of valuable methods are proposed [4,5,6].

The conventional initial alignment procedure is usually divided into two phases [7,8]. The first phase is called the coarse alignment process, where an analytical method is utilized to accomplish this phase. In the coarse alignment process, the large misalignment angles between the body and navigation frames are roughly acquired, the horizontal misalignment angles will be less than one degree, and the heading misalignment angle is constrained in several degrees [9,10]. The second phase is the fine alignment process [11], and the nonlinear error models of strapdown inertial navigation can be approximated to the linear error models due to the small misalignment angles, which are acquired by the coarse alignment. In this phase, the sensor’s constant bias noises are estimated by a linear Kalman filter and the misalignment angles can be further reduced. Moreover, the horizontal errors and heading errors are less than 0.01 degree and 0.1 degree, respectively. Therefore, the coarse alignment is a fundamental of the traditional fine alignment, and the coarse alignment with high performance will increase the convergence rate of the fine alignment, and the total alignment time can be made shorter [12,13].

The general coarse alignment method can be divided into two categories according to different observation vectors. One takes advantage of the apparent gravity to determine the initial attitude of the SINS [1,14], and the other is based on apparent velocity [10,15]. Currently, the coarse alignment method based on apparent velocity is widely applied due to the smoothed properties of the apparent velocity, and an optimization-based method based on Wahba’s problem becomes very popular [16,17,18,19]. In this coarse alignment method, the initial coarse alignment procedure had been transferred into an attitude determination method, and the optimal quaternion was determined by a least-squares method. Then the time-varying attitude of SINS can be calculated by the chain rule of the determined direction cosine matrix (DCM). However, there are two major problems in the apparent velocity method; the first one is that the alignment errors will drift with time due to the integration of the inertial sensors’ outputs. The second one is that the performance of the coarse alignment will be poor without an additional reference velocity when the linear velocity perturbation exists. These two defects of the apparent velocity method limit its applications in practical situations. Hence, if the interference of the apparent gravity can be eliminated from the measurement observation vectors, it will be a good choice for coarse alignment in some special applications, such as in-motion self-contained coarse alignment.

Different from the apparent velocity method is the apparent gravity method, where the outputs of an accelerometer are employed to construct the measurement observation vectors directly, and where the integration process is not needed in this method so that the alignment errors will not drift with time. Moreover, when the initial position of the carrier is well known, the apparent gravity will be computed precisely, and it is independent with the carrier’s movements. Based on these features, the coarse alignment will be carried out whenever there is interference of the unknown velocity of the carriers, and the precision of the coarse alignment during the whole alignment procedure can be improved [20,21]. However, there is a drawback with this method, which is that the apparent gravity is submerged by the external acceleration of the vehicle movements, so it is hard to determine the misalignment angles according to inertial outputs straightforwardly. In order to address this issue, a low-pass digital filter is designed for filtering out the high-frequency noises, which distort the effective apparent gravity [22,23]. However, it is difficult to design a suitable low-pass digital filter for practical applications, because the external environment is inconstant and the movements of the carrier cannot be completely eliminated by the digital filter. These complicated noises still exist in the observation vectors, which is why the development of the coarse alignment based on the apparent gravity method is hindered.

In [14], a parameter recognition method is developed for coarse alignment based on apparent gravity. However, it can only be applied in a swaying base case. Inspired by digital filter methods, a new coarse alignment algorithm based on apparent gravity method is investigated. Considering the in-motion coarse alignment, firstly, an infinite impulse response (IIR) low-pass is designed in this paper for filtering out the high frequency, which is resulting from the engine noises and external disturbation. Then, a parameter recognition and new reconstruction algorithm for extracting the apparent gravity, which is used to calculate the observation vectors, is developed. Finally, in order to address the outlier in the measurement observation vectors, a robust filter based on Huber’s M-estimation theory is designed. All of the improved methods are verified by the designed tests in detail.

This paper is organized as follows: the definitions of the reference frames in this paper are introduced in the next section. In Section 3, the general mechanisms of the coarse alignment method based on apparent gravity are summarized, and the observation vectors are defined simultaneously. In Section 4, an IIR low-pass digital filter is designed, and the power spectrum of the measurement observation vectors is analyzed. The parameter recognition and observation vectors reconstructed methods are developed in Section 5. To show the performance of the proposed method, the simulation, turntable and vehicle tests are designed in Section 6. Finally, the conclusions of this paper are summarized in Section 7.

## 2. Definition of Coordinate Frame

The coordinate frames used in this paper are defined as follows:

1. *i*-frame: Earth-centered initially-fixed orthogonal reference frame;
2. *n*-frame: orthogonal reference frame aligned with East-North-Up (ENU) geodetic axes;
3. *n*0-frame: orthogonal reference frame non-rotating relative to the *i*-frame, which is formed by fixing the *n*-frame at start up in the inertial space;
4. *b*-frame: orthogonal reference frame aligned with IMU axes;
5. *b*0-frame: orthogonal reference frame non-rotating relative to the *i*-frame, which is formed by fixing the *b*-frame at start up in the inertial space;
6. *e*-frame: Earth-centered Earth-fixed (ECEF) orthogonal reference frame;
7. *e*0-frame: orthogonal reference frame non-rotating relative to the *i*-frame, which is formed by fixing the *e*-frame at start up in the inertial space.

**Figure 1:**
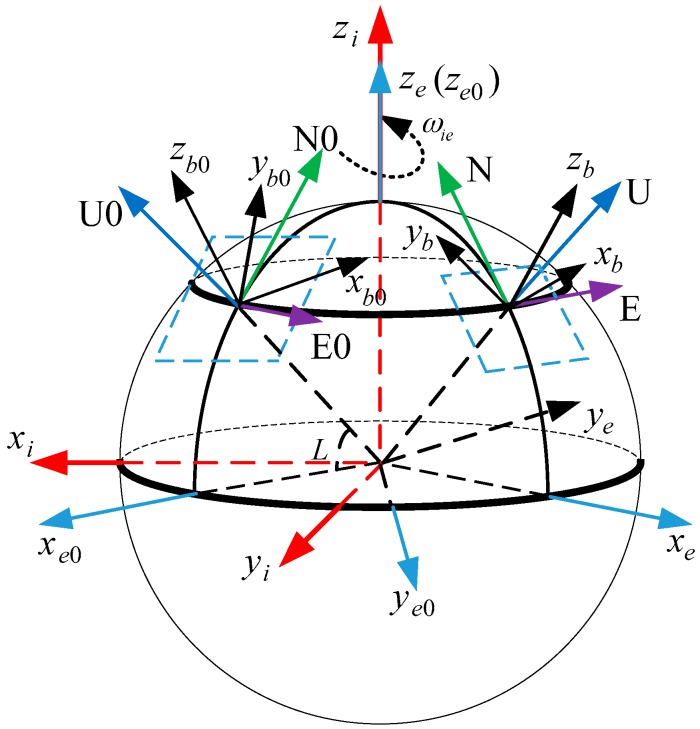
The definition of the coordinate frames.

Figure 1 illustrates the coordinate frames.

## 3. Mechanisms of Inertial Frame Alignment Based on Apparent Gravitational Motion

It is well known that the main purpose of the SINS initial alignment is to obtain the misalignment angles of the *b*-frame with respect to the *n*-frame, and the DCM is described as $C_{b}^{n}$. If the carrier is in the stationary case, the DCM $C_{b}^{n}$ at time instant *t* is equal to that at start up, and it can be calculated easily by the Earth rate and the local gravity. However, in the practical situation, the carrier is not always in the stationary state, such as the swaying case and the in-motion case, so the misalignment angles are time-varying. Additionally, the Earth rate and the local gravity are contaminated in these situations, and it is difficult to calculate the DCM $C_{b}^{n}$ at time instant *t* straightforwardly according to the inertial sensors’ outputs. To address this issues, the matrix $C_{b}^{n}$ has been divided into three matrices by the chain rule of the DCM:

(1) $$C_{b\left(t\right)}^{n\left(t\right)}=C_{n0}^{n\left(t\right)}C_{b0}^{n0}C_{b\left(t\right)}^{b0}$$

where

(2) $$\{\begin{array}{c} {\dot{C}}_{b\left(t\right)}^{b0}=C_{b\left(t\right)}^{b0}\left[\omega_{ib}^{b}\times \right]\\ {\dot{C}}_{n\left(t\right)}^{n0}=C_{n\left(t\right)}^{n0}\left[\omega_{in}^{n}\times \right] \end{array}$$

and, $C_{b\left(t\right)}^{b0}$ and $C_{n\left(t\right)}^{n0}$ are the identity matrix at start up, and they can be calculated by the iteration operations using $\omega_{ib}^{b}$ and $\omega_{in}^{n}$, respectively [10].

According to the aforementioned analysis, once the DCM $C_{b0}^{n0}$ is known, the DCM $C_{b\left(t\right)}^{n\left(t\right)}$ can be calculated by Equation (1), and the coarse alignment process will end. Furthermore, it is noted that the matrix $C_{b0}^{n0}$ is constant during the whole alignment process, and this characteristic is helpful to improve the precision of the extracted misalignment angles at start up according to some parameters recognition methods.

The apparent velocity update equation in the *n*-frame is known as

(3) $${\dot{v}}^{n}=f^{n}-\left(2\omega_{ie}^{n}+\omega_{en}^{n}\right)\times v^{n}+g^{n}.$$

According the matrix multiplication, a three-component vector can be transformed from *n*-frame to the *b*-frame:

(4) $$f^{n}=C_{b\left(t\right)}^{n\left(t\right)}f^{b}.$$

Substituting Equations (1) and (4) into Equation (3) yields

(5) $$C_{b\left(t\right)}^{b0}f^{b}-C_{b\left(t\right)}^{b0}C_{n\left(t\right)}^{b\left(t\right)}{\dot{v}}^{n}-C_{b\left(t\right)}^{b0}C_{n\left(t\right)}^{b\left(t\right)}\left(2\omega_{ie}^{n}+\omega_{en}^{n}\right)\times v^{n}=-C_{n0}^{b0}C_{n\left(t\right)}^{n0}g^{n}.$$

Defining the reference vector and the observation vector as

(6) $$\{\begin{array}{c} \alpha =-C_{n\left(t\right)}^{n0}g^{n}\;\\ \beta =C_{b\left(t\right)}^{b0}\left(f^{b}-C_{n\left(t\right)}^{b\left(t\right)}\left({\dot{v}}^{n}+\left(2\omega_{ie}^{n}+\omega_{en}^{n}\right)\times v^{n}\right)\right) \end{array}.$$

Equation (5) can be rewritten as the vector observations-based measurement model for $C_{b0}^{n0}$ as

(7) $$\beta =C_{n0}^{b0}\alpha .$$

**Figure 2:**
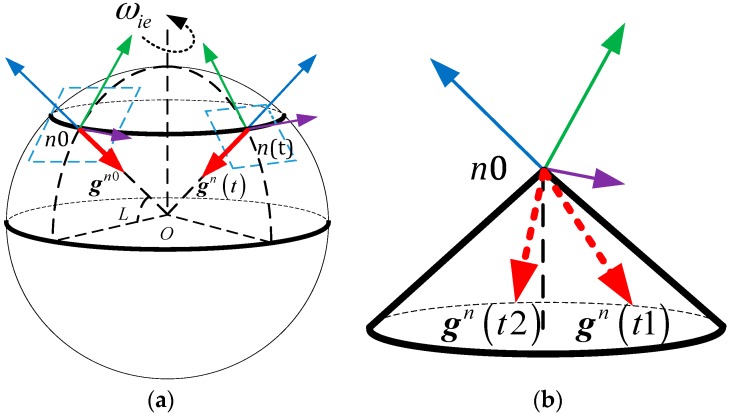
(**a**) The *n*-frame motion due to the self-rotation of the earth; (**b**) the apparent gravitational motion in the *n*0-frame.

Figure 2 shows mechanisms of inertial frame alignment based on apparent gravitational motion. Figure 2a describes the motion of the *n*-frame due to the self-rotation of the Earth during the coarse alignment procedure, where the left orthogonal reference frame represents the *n*0-frame and the right orthogonal reference frame represents the *n*-frame at time instant *t*. In the two orthogonal frames, the purple arrow, the green arrow, and the blue arrow represent east, north, and up axes of the *n*-frame, respectively.

The apparent gravitational motion in the *n*0-frame is shown in Figure 2b, and it can be found that any two non-collinear vectors, which are projected on the *n*0-frame, can be obtained at two different time instants. Then, based on the three-axis attitude determination (TRIAD) algorithm [9], the matrix $C_{n0}^{b0}$ can be calculated by

(8) $$C_{b\left(0\right)}^{n\left(0\right)}={\left[\begin{array}{c} \frac{\alpha^{T}\left(t1\right)}{∥\alpha^{T}\left(t1\right)∥}\\ \frac{{\left(\alpha \left(t1\right)\times \alpha \left(t2\right)\right)}^{T}}{∥{\left(\alpha \left(t1\right)\times \alpha \left(t2\right)\right)}^{T}∥}\\ \frac{{\left(\alpha \left(t1\right)\times \alpha \left(t2\right)\times \alpha \left(t1\right)\right)}^{T}}{∥{\left(\alpha \left(t1\right)\times \alpha \left(t2\right)\times \alpha \left(t1\right)\right)}^{T}∥} \end{array}\right]}^{-1}\left[\begin{array}{c} \frac{\beta^{T}\left(t1\right)}{∥\beta^{T}\left(t1\right)∥}\\ \frac{{\left(\beta \left(t1\right)\times \beta \left(t2\right)\right)}^{T}}{∥{\left(\beta \left(t1\right)\times \beta \left(t2\right)\right)}^{T}∥}\\ \frac{{\left(\beta \left(t1\right)\times \beta \left(t2\right)\times \beta \left(t1\right)\right)}^{T}}{∥{\left(\beta \left(t1\right)\times \beta \left(t2\right)\times \beta \left(t1\right)\right)}^{T}∥} \end{array}\right]$$

where ∥·∥ represents the normalized operation. If the observation vector β can be acquired accurately, the DCM $C_{b\left(0\right)}^{n\left(0\right)}$ can be obtained precisely. Thus, how to extract the effective observation vector $\hat{\beta }$ from the measurement $\tilde{\beta }$ is essential in the inertial frame coarse alignment. In the next sections, some methods for extracting $\hat{\beta }$ will be introduced in detail.

## 4. IIR Low-Pass Digital Filter

According to the aforementioned analysis, the accuracy of the extracted observation vector $\hat{\beta }$ is of vital importance to the coarse alignment. However, it is submerged in complex noises of the external environment, and it is difficult to compute the initial attitude directly by the measurements of the inertial sensors. In this section, we will analyze the noise components of the measurement observation vector $\tilde{\beta }$, and then an IIR low-pass digital filter will be designed to eliminate the high-frequency noises.

### 4.1. Measurement Model of the Observation Vector

In this work, the measurement models of the inertial sensors are defined by

(9a) $${\tilde{f}}^{b}=f^{b}+{𝛻}^{b}+\epsilon^{b}$$

(9b) $${\tilde{\omega }}^{b}=\omega^{b}+\varepsilon^{b}+\eta^{b}$$

where ${\tilde{f}}^{b}$ is the actual output of the accelerometer; ${𝛻}^{b}$ and $\epsilon^{b}$, respectively, denote the constant bias and random noise; ${\tilde{\omega }}^{b}$ is the measured angular velocity of the *b*-frame with respect to the *i*-frame; $\varepsilon^{b}$ and $\eta^{b}$ denote the constant bias and random noises in the IMU axes.

According to the measurement models of the inertial sensors, the measurement model of the observation vector can be calculated by

(10) $$\tilde{\beta }={\tilde{C}}_{b\left(t\right)}^{b0}{\tilde{f}}^{b}$$

where ${\tilde{C}}_{b\left(t\right)}^{b0}$ is calculated by Equation (2) using the angular velocity ${\tilde{\omega }}^{b}$.

Let $\delta C_{b\left(t\right)}^{b0}$ represents the error matrix between ${\tilde{C}}_{b\left(t\right)}^{b0}$ and $C_{b\left(t\right)}^{b0}$:

(11) $${\tilde{C}}_{b\left(t\right)}^{b0}=C_{b\left(t\right)}^{b0}+\delta C_{b\left(t\right)}^{b0}.$$

Substituting Equations (11) and (9a) into Equation (10) yields

(12) $$\tilde{\beta }=\left(C_{b\left(t\right)}^{b0}+\delta C_{b\left(t\right)}^{b0}\right)\left(f^{b}+{𝛻}^{b}+\epsilon^{b}\right).$$

Through some calculation operations, Equation (12) can be expressed as

(13) $$\tilde{\beta }=\beta +{\tilde{C}}_{b\left(t\right)}^{b0}C_{n\left(t\right)}^{b\left(t\right)}\left({\dot{v}}^{n}+\left(2\omega_{ie}^{n}+\omega_{en}^{n}\right)\times v^{n}\right)-\delta C_{b\left(t\right)}^{b0}C_{n\left(t\right)}^{b\left(t\right)}g^{n}+C_{b\left(t\right)}^{b0}\left({𝛻}^{b}+\epsilon^{b}\right).$$

where the second-order errors have been removed. Due to the short time of the coarse alignment process and the relative slow moving velocity of the carrier, the term ${\tilde{C}}_{b\left(t\right)}^{b0}C_{n\left(t\right)}^{b\left(t\right)}\omega_{en}^{n}\;\times \;v^{n}$ is much smaller [22,23,24], so that it can be ignored from the measurement model. The magnitude of term $2{\tilde{C}}_{b\left(t\right)}^{b0}C_{n\left(t\right)}^{b\left(t\right)}\omega_{ie}^{n}\times v^{n}$ is equivalent to the magnitude of the constant bias $C_{b\left(t\right)}^{b0}{𝛻}^{b}$, so it does not have much influence on the alignment results. Within these assumptions, Equation (13) can be simplified as

(14) $$\tilde{\beta }=\beta +{\tilde{C}}_{b\left(t\right)}^{b0}C_{n\left(t\right)}^{b\left(t\right)}{\dot{v}}^{n}+\delta^{b0}+\varsigma^{b0}$$

where

(15) $$\{\begin{array}{c} \varsigma^{b0}=-\delta C_{b\left(t\right)}^{b0}C_{n\left(t\right)}^{b\left(t\right)}g^{n}+C_{b\left(t\right)}^{b0}\epsilon^{b}\\ \delta^{b0}=C_{b\left(t\right)}^{b0}{𝛻}^{b}+2{\tilde{C}}_{b\left(t\right)}^{b0}C_{n\left(t\right)}^{b\left(t\right)}\omega_{ie}^{n}\times v^{n} \end{array}.$$

Additionally, $\varsigma^{b0}$ is a high-frequency noise, which is comprised with the engine noise, movement noise and inertial sensor noise. $\delta^{b0}$ denotes the constant bias of the observation vectors.

### 4.2. IIR Low-Pass Digital Filter for the High-Frequency Noises

Based on the measurement observation vector in Equation (14), it can be found that the measurement noises are complex, requiring specific methods for different noises to be designed. In this subsection, we devote to filtering the high-frequency noises of the measurement observation vectors.

**Figure 3:**
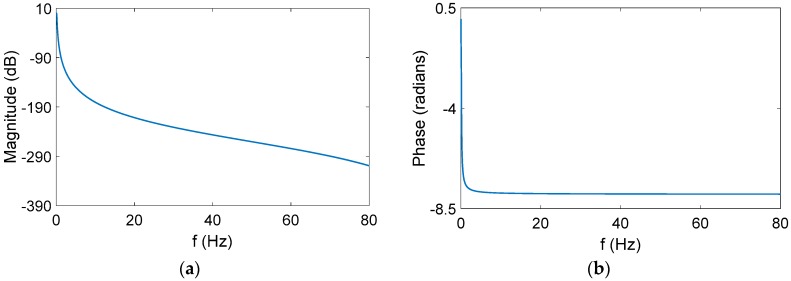
(**a**) The curves of amplitude-frequency response; (**b**) curves of the phase-frequency response.

In [23], an FIR digital filter has been designed for extracting the true observation vectors from the measurement observation vectors. Considering that the passband of the filter is very small, the FIR filter orders are very high, and it is difficult to apply in the practical systems. To address this defect, we choose an IIR low-pass digital filter for our applications. By using the “fadtool” in MATLAB 2014a (MathWorks, Inc., Natick, MA, USA), the special parameters, such as sampling rate, desirable passband and stopband attenuation, and the passband and stopband frequencies, are input in the interactive interface straightforwardly, so that the IIR low-pass digital filter is easily designed, and the transfer function of the designed digital filter is also acquired. The parameters of the filter are listed in Table 1, and the corresponding curves of the amplitude-frequency response and phase-frequency response are depicted in Figure 3. In Table 1, Apass denotes the desirable passband attenuation, Astop denotes the desirable stopband attenuation, and Fpass and Fstop are the passband and stopband frequencies, respectively. Fs represents the sampling rate of the measurement observation vectors.

After some manipulations, the IIR filter transfer function can be simplified as

(16) $$H(z)=\frac{0.002496-0.007435z^{-1}+0.004887z^{-2}+0.004991z^{-3}-0.007383^{-4}+0.002444z^{-5}}{1-4.983886z^{-1}+9.935674z^{-2}-9.903705z^{-3}+4.935932z^{-4}-0.984012z^{-5}}$$

**Figure 4:**
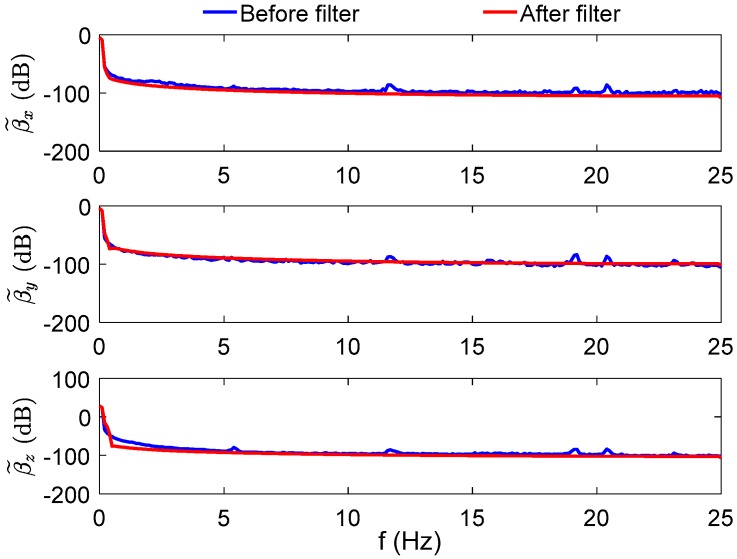
The power spectrum of the observation vector.

To confirm our above analysis, vehicle trial data has been filtered out by the designed digital filter, and the power spectrum of the measurement observation vectors is shown in Figure 4, where the red line denotes the filtered measurement observation vectors, and the blue line represents the original measurement observation vectors.

It can be obviously found that the frequency of the apparent gravitational vectors are much lower than acceleration disturbances, which are engine noises, variable motion, sensors noises, and so on, when the vehicle is in motion. The effective frequency of the varying measurement observation vectors is lower than 0.1 Hz. By using the designed digital filter, the lower frequency part of the measured observation vectors is retained, and the high-frequency part has been filtered out.

The low-pass digital filter is helpful to eliminate the high interferential frequency noises from the observation vectors, but there are other interfering noises remaining in the extracted vectors, such as phase shift interference, spectrum leakage, etc. As known, these defects will degrade the precision of the extracted observation vectors, and they can also lead to the poor performance of coarse alignment. To solve these problems, a parameter recognition and observation vector reconstruction method will be proposed in the ensuing Section 5.

## 5. Parameter Recognition and Observation Vector Reconstruction

In this section, a novel method to extract the apparent gravitational motion from the observation vectors, which has been filtered by the IIR filter, will be introduced. According to the analysis of the features of the apparent gravitational motion, a parameter model has been investigated, and the corresponding recognition algorithm is developed. Based on the optimal parameters, the reconstructed algorithm of the observation vectors will be developed, which can enlarge the non-collinearity of the two observation vectors, and this is helpful to improve the performance of alignment.

### 5.1. Parameter Model of Apparent Gravity

According to the chain rule of the DCM, Equation (7) can be rewritten as

(17) $$\beta =-C_{e0}^{b0}C_{e\left(t\right)}^{e0}C_{n\left(t\right)}^{e\left(t\right)}g^{n}$$

where $C_{e0}^{b0}$ is a constant matrix during the whole alignment process. Due to the short time of the alignment process and the low velocity of the vehicle, the DCM $C_{n\left(t\right)}^{e\left(t\right)}$ can be approximated to a constant matrix.

As aforementioned analysis, Equation (17) can be expanded as

(18) $$\beta =C_{n\left(0\right)}^{b\left(0\right)}\left[\begin{array}{ccc} 0 & g\cos\left(L\right) & 0\\ -g\sin\left(L\right)\cos\left(L\right) & 0 & g\sin\left(L\right)\cos\left(L\right)\\ g\cos^{2}\left(L\right) & 0 & g\sin^{2}\left(L\right) \end{array}\right]\left[\begin{array}{c} \cos\left(\omega_{ie}t\right)\\ \sin\left(\omega_{ie}t\right)\\ 1 \end{array}\right]$$

where $\omega_{ie}$ is the Earth rate, L denotes the geographic latitude, and g represents the magnitude value of gravity on local latitude. The detail calculation of Equation (18) can be referred to Appendix A. Then, the parameter model is given by

(19) $$\beta =\left[\begin{array}{ccc} \gamma_{11} & \gamma_{12} & \gamma_{13}\\ \gamma_{21} & \gamma_{22} & \gamma_{23}\\ \gamma_{31} & \gamma_{32} & \gamma_{33} \end{array}\right]\left[\begin{array}{c} \cos\left(\omega_{ie}t\right)\\ \sin\left(\omega_{ie}t\right)\\ 1 \end{array}\right]$$

where $\gamma_{ij}\left(i=1,2,3;j=1,2,3\right)$ indicates the unknown constant value.

Let $\bar{\beta }$ denotes the filtered observation vector. Since the ideal parameter model has been constructed, the actual discrete model can be easily given by

(20) $$\{\begin{array}{c} \gamma_{k+1}=\gamma_{k}\\ {\bar{\beta }}_{k+1}=\gamma_{k+1}M_{k+1}+\varrho_{k+1} \end{array}$$

where $M_{k+1}={\left[\cos\left(\omega_{ie}t_{k+1}\right)\;\sin\left(\omega_{ie}t_{k+1}\right)\;1\right]}^{T}$, and $\varrho_{k+1}$ denotes the unknown noises, of which the covariance is uncertain. In the next subsection, a parameter recognition algorithm will be designed, and the parameters will be estimated by the developed Kalman filter.

### 5.2. Parameter Recognition Based on the Adaptive Kalman Filter

According to the analysis above, the statistic information of the measurement noise $\varrho_{k+1}$ is unknown, because observation vector $\tilde{\beta }$ has been filtered by the designed low-pass digital filter and contains the unknown disturbances. Thus, the traditional RLS algorithm cannot be used to estimate the parameters, because the precise covariance of the measurement noise is necessary to this method. Based on [14], a linear Kalman filter based on adaptive technology has been investigated for addressing the uncertainty measurement noises.

Since the three elements of the observation vectors are uncorrelated, the parameter model can be divided into three independent forms, which can be conducted by the familiar estimated procedure. Here, the particular simplified model of the third component of ${\bar{\beta }}_{k+1}$ is given by

(21) $$\{\begin{array}{c} \gamma_{z,k+1}=\gamma_{z,k}\\ {\bar{\beta }}_{z,k+1}=\gamma_{z,k+1}M_{k+1}+\varrho_{z,k+1} \end{array}.$$

The Kalman filter for Equation (21) is summarized as follows:

(22) $$e_{z,k+1}={\bar{\beta }}_{z,k+1}-{\hat{\gamma }}_{z,k}M_{k+1}$$

(23) $$\Lambda_{z,k+1}=\Lambda_{z,k}+\frac{1}{k+1}\left[e_{z,k+1}e_{z,k+1}^{T}-\Lambda_{z,k}\right]$$

(24) $$G_{k+1}=P_{z,k}M_{k+1}{\left[M_{k+1}^{T}P_{z,k}M_{k+1}+\Lambda_{z,k+1}\right]}^{-1}$$

(25) $${\hat{\gamma }}_{z,k+1}={\hat{\gamma }}_{z,k}+G_{k+1}^{T}e_{z,k+1}$$

(26) $$P_{z,k+1}=P_{z,k}-G_{k+1}\left[M_{k+1}^{T}P_{z,k}M_{k+1}+\Lambda_{z,k+1}\right]G_{k+1}^{T}$$

where $\Lambda_{z,k+1}$ is an estimated covariance of the filtered measurement noise based on k+1 data pairs, and the term $e_{z,k+1}$ is the objective function for the measurement residual process.

In the estimation process, the covariance of the measurement noise is estimated in real-time, and the estimated value is compensated by the algorithm. Thus, the adaptive performance of the algorithm will be enhanced. Although the Kalman filter can suppress the unknown noises, it will be poor when the measurement contains the outliers, which is caused by the irregular maneuver of the vehicle. This interference will degrade the performance of the Kalman filter or even make the filter diverging. This problem will be addressed in Section 5.3.

### 5.3. Robust Filter

From Equation (14), we can find that the outliers will be generated when the vehicle is in random motion or uniform variable motion and, if the vehicle is in uniform rotation motion, there is radial acceleration in the measurement observation vectors. These disturbances cannot be easily eliminated by an optimal state estimation method; a common method to address the outliers of the measurements is the robust estimation method. For the engineering application, the Huber’s M-estimation is a practical method. In this work, a robust Kalman filter based on M-estimation has been designed for suppressing the aforementioned noises. 

The M-estimation entails minimizing the criterion function of the form:

(27) $$J_{M}=\overset{n}{\underset{i=1}{\sum }}\rho \left({\left|{\bar{\beta }}_{k+1}-{\hat{\gamma }}_{k}M_{k+1}\right|}_{i}\right)$$

where ${\left|\cdot \right|}_{i}$ denotes the *i*th element of the vector, and ρ(·) denotes a less rapidly increasing function than the square. This ensure that large residual errors, which correspond to outliers, do not influence the optimization of $J_{M}$ [25]. It is noted that, if ρ(·) equals the square function, the standard least squares criterion has been obtained [26]. Furthermore, the following special case based on weighted least squares criterion can be obtained:

(28) $$J_{M}={\left({\bar{\beta }}_{k+1}-{\hat{\gamma }}_{k}M_{k+1}\right)}^{T}S\left({\bar{\beta }}_{k+1}-{\hat{\gamma }}_{k}M_{k+1}\right)$$

where S is a diagonal matrix, the diagonal entries $S_{i,i}$ determine the weight accorded to the corresponding data residual ${\left|{\bar{\beta }}_{k+1}-{\hat{\gamma }}_{k}M_{k+1}\right|}_{i}$. A simple but attractive choice for these weights is the non-linear function given by

(29) $$S_{i,i}=\frac{1}{\Lambda_{i,k+1}}min\left\{1,\frac{c_{i}}{{\left|{\bar{\beta }}_{k+1}-{\hat{\gamma }}_{k}M_{k+1}\right|}_{i}^{2}}\right\}$$

where $c_{i}$ is the *i*th threshold parameter that can be modulated by the practical application. In order to understand the performance of the function, it is noted that S effectively clips the *i*th value in $J_{M}$ to a constant value $c_{i}$ when the *i*th squared residual ${\left|{\bar{\beta }}_{k+1}-{\hat{\gamma }}_{k}M_{k+1}\right|}_{i}^{2}$ exceeds the threshold $c_{i}$; otherwise, the value is set equal to the squared residual.

According to the above analysis, the update steps of the adaptive Kalman filter can be re-derived as

(30) $${\hat{G}}_{k+1}=\left[M_{k+1}D_{z,k}M_{k+1}^{T}+P_{z,k}^{-1}\right]{\;}^{-1}M_{k+1}D_{z,k}$$

(31) $${\hat{\gamma }}_{z,k+1}={\hat{\gamma }}_{z,k}+{\hat{G}}_{k+1}^{T}e_{z,k+1}$$

(32) $$P_{z,k+1}=P_{z,k}-{\hat{G}}_{k+1}\left[M_{k+1}^{T}P_{z,k}M_{k+1}+\Lambda_{z,k+1}\right]{\hat{G}}_{k+1}^{T}$$

where

(33) $$D_{z,k}=\{\begin{array}{cc} 0, & \;\mathrm{if}\;{\left|{\bar{\beta }}_{k+1}-{\hat{\gamma }}_{k}M_{k+1}\right|}_{z}^{2}>c_{z}\\ \frac{1}{\Lambda_{z,k+1}}, & \mathrm{otherwise} \end{array}$$

where $D_{z,k}$ can be regarded as the sensory residual gain or “gating” scale, which determines the gain on the incoming sensory residual errors, the detailed derivation of robust filter can be referred to Appendix B. By effectively filtering out any high residuals, $D_{z,k}$ allows the Kalman filter to ignore the corresponding outliers in the input ${\bar{\beta }}_{k+1}$, thereby enabling it to robustly estimate the state ${\hat{\gamma }}_{z,k+1}$. According to the above method, the term ${\tilde{C}}_{b\left(t\right)}^{b0}C_{n\left(t\right)}^{b\left(t\right)}{\dot{v}}^{n}$ of Equation (13) can be eliminated from the observation vectors effectively, the apparent gravitational motion is extracted from the measurements precisely.

### 5.4. Observation Vectors Reconstruction

Since the optimal parameters of the apparent gravity has been extracted from above methods, the effective observation vectors can be constructed by Equation (19), which are named the reconstructed observation vectors. Nevertheless, there is another issue, which is that the collinear properties of the observation vectors at two different times must be addressed. According to Equation (19), the reconstructed observation vectors at time instant k+1 is given by

(34) $${\hat{\beta }}_{k+1}={\hat{\gamma }}_{k+1}\left[\begin{array}{c} \cos\left(\omega_{ie}\left(k+1\right)∆t\right)\\ \sin\left(\omega_{ie}\left(k+1\right)∆t\right)\\ 1 \end{array}\right]$$

where ∆t is the sampling period of the inertial sensors. Due to the constant features of the parameter matrix, ${\hat{\gamma }}_{k+1}$ is just correlated with the initial attitude at start up and the vehicle position, which can be shown in Equation (18). Once the alignment starting, it is a constant matrix during the whole alignment process, and the apparent gravitational motion is just correlated with the Earth rotation. In this work, to enlarge the non-collinear properties of the two reconstructed observation, ${\hat{\beta }}_{k+1}$ and ${\hat{\beta }}_{0}$ are chosen as the dual-vectors, where

(35) $${\hat{\beta }}_{0}={\hat{\gamma }}_{k+1}\left[\begin{array}{c} 1\\ 0\\ 1 \end{array}\right].$$

**Figure 5:**
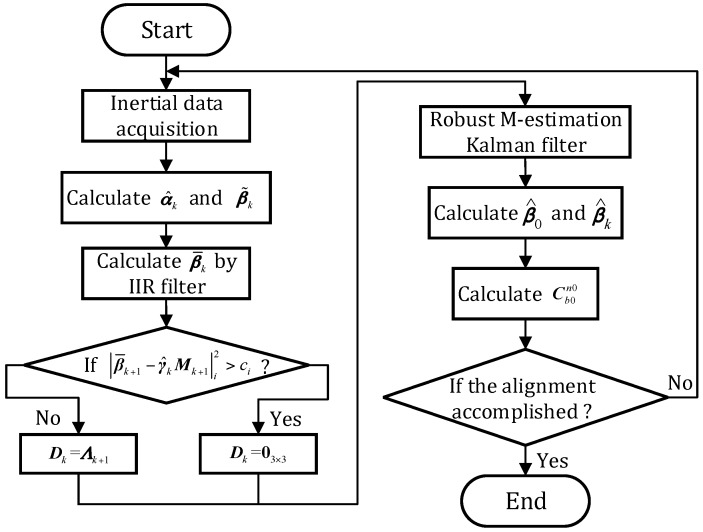
The flow diagram of algorithm.

Consequently, the entire procedure of the novel coarse alignment algorithm has been established. For more clarity, the flow diagram of algorithm is shown in Figure 5.

## 6. Simulation and Physical Tests

In this work, we considered the practical applications of our method, thus the simulation and physical tests were designed to verify the algorithm. The experiments are processed in two cases, which are the swaying case and the in-motion case. Three traditional methods, which are applied in practical systems, are designed for comparison. They are as follows: (i) the traditional apparent gravitational method, which is represented as Scheme 1 [20]; (ii) the digital filter method, which is represented as Scheme 2 [23]; and (iii) the in-motion method based on the apparent velocity, which is denoted as Velocity Integration Formula (VIF) method [18]. The proposed method in this paper is denoted as Scheme 3.

### 6.1. Simulation Tests on the Swaying Base

In this subsection, a simulation test for the swaying base is studied. The sawing rule is Asin(2πft+φ)+θ, and A and f are the amplititude and frequency of the swaying motion, while φ and θ represent the initial phase and swaying center, respectively. The swaying parameters for the test are listed in Table 2.

The whole coarse alignment of this test lasts for 600 s, and the geographic latitude and longitude of the vehicle are set as L=32° and λ=118°. By using the aforementioned parameters, the outputs of the inertial sensors can be collected by the back-stepping algorithm of the SINS solution. Then, the coarse alignment results can be calculated by the generated outputs of the inertial sensors. In this simulation, the sensor errors are set in Table 3.

**Figure 6:**
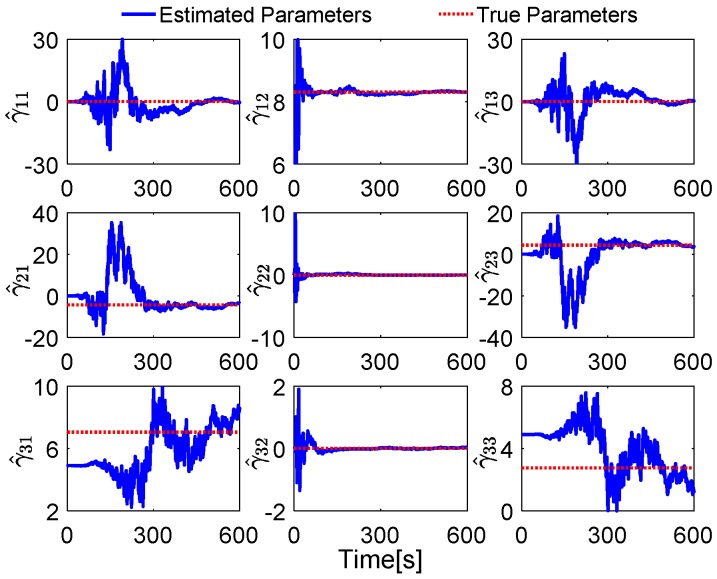
Comparison between estimated parameters and true parameters.

**Figure 7:**
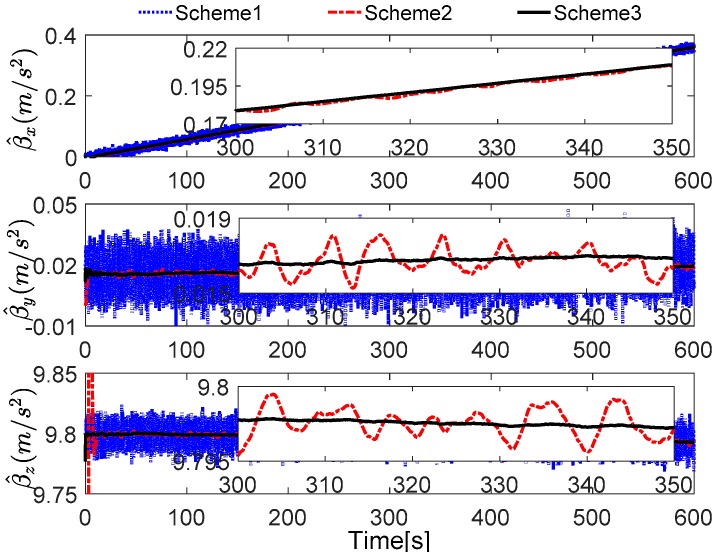
Curves of the reconstructed observation vectors.

**Figure 8:**
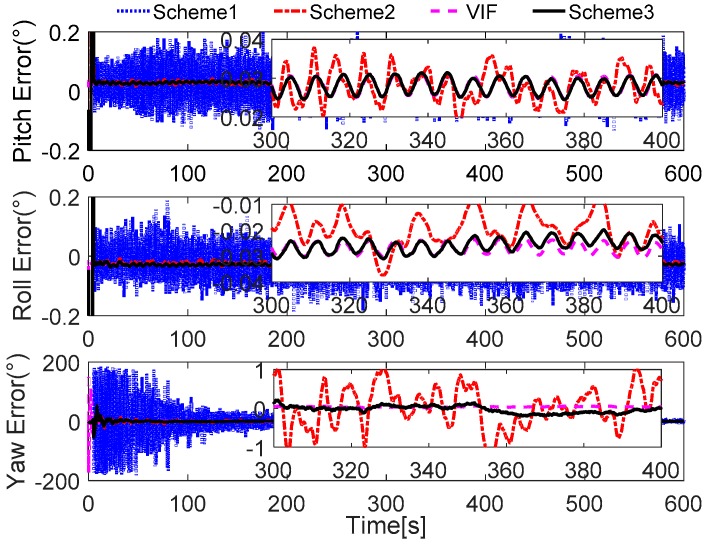
Curves of alignment errors.

Without loss of generality, the initial parameters matrix ${\hat{\gamma }}_{0}$ is set as a 3 × 3 null matrix, and the initial estimation error covariance matrix is set as $P_{i,0}=diag\left[10,000\;10,000\;10,000\right]$, and the adaptive measurement noise is set as $\Lambda_{i,0}=0.1$. The robust filter threshold $c_{i}$ is equal to 0.05 in the simulation test. The simulation results of the swaying motion are shown in Figure 6, Figure 7 and Figure 8, and the estimated parameters matrix $\hat{\gamma }$ is shown in Figure 6. The observation vectors, which are employed to different methods, are depicted in Figure 7, while the alignment errors of four methods are shown in Figure 8.

In Figure 6, the red dotted line represents the true parameters and the blue line denotes the estimated parameters. Since the initial attitude is set as ${\left[0\;0\;0\right]}^{T}$ at start up, the DCM $C_{n\left(0\right)}^{b\left(0\right)}$ is equal to the 3 × 3 identity matrix, and the true parameter matrix is shown in Equation (18). It can be found that the second parameters of each row of $\hat{\gamma }$ have a faster convergence rate, while the first and the third ones have wide fluctuation; these features are reasonable because the second element in each row of $\hat{\gamma }$ is the main components of the reconstructed observation vectors, and the small components are consisted with the first and the third elements in each row of $\hat{\gamma }$, when the initial attitude is set as ${\left[0\;0\;0\right]}^{T}$ at start up. 

In Figure 7, the reconstructed observation vectors of Scheme 3 has confirmed our aforementioned analysis, and they are smoothed. Due to the existing noises and the defects of the digital filter, the observation vectors of Scheme 1 and Scheme 2 are fluctuant, but it also can be notes that many high-frequency noises have be eliminated in Scheme 2. These different features will influence the performance of coarse alignment directly.

In Figure 8, it is shown that the alignment results of Scheme 3 are more stable than Scheme 1 and Scheme 2, and are equivalent to the current VIF method, which is based on apparent velocity. For convenient comparison, the partial enlarged views of the alignment errors between 300 s and 400 s are depicted, where the errors of Scheme 1 have been ignored due to the wide fluctuation. 

To verify our analysis, the statistics of three methods between 300 s and 400 s are listed in Table 4. It can be obviously found that the mean values of the errors of horizontal angles are closer, which are around 0.028° for pitch error and −0.025° for roll error. However, as the STD value shows, the horizontal angles of three methods error of Scheme 3 and VIF method are around 0.0020°, while the horizontal angles error of Scheme 2 is greater than 0.004°, which is more than twice as much as the other two methods. In the yaw errors, the same features can be found. This reveals that the results of Scheme 2 are unstable, and the performance of Scheme 2 will decline in the harsh external environment. It can be also noted that the STD value of yaw errors of Scheme 3 is 0.0904°, while that of the VIF method is 0.0098°, which is much smaller than Scheme 3. This is because the apparent gravity are more sensitive to the external noises than the apparent velocity, and the simulation condition are ideal. In the practical cases, there always exist linear velocity disturbation, then Scheme 3 will be better than the VIF method. This is verified in the next subsections.

### 6.2. Simulation Tests for the Vehicle Motion

**Figure 9:**
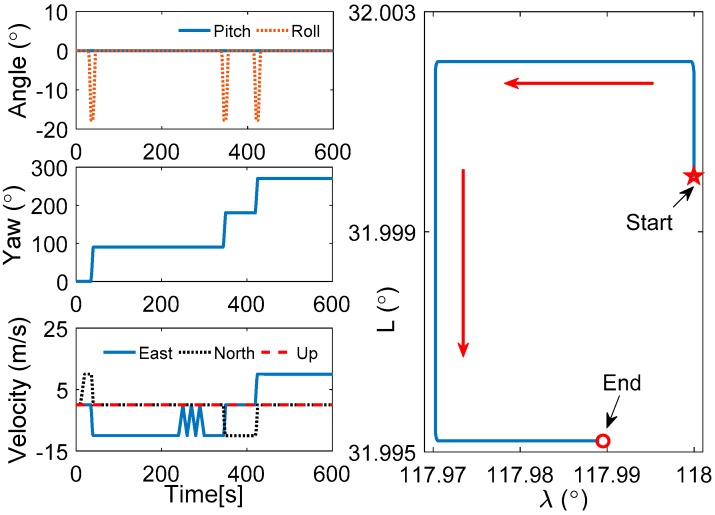
Curves of vehicle motion.

In the swaying simulation, it is shown that the performance of Scheme 3 is better than Scheme 2, and it is equivalent to the VIF method. In this work, the application of the initial alignment under the in-motion base is considered, and the simulation for the vehicle test is designed in this subsection. In Table 5, the state of the vehicle motion is listed, the curves of the vehicle motion are depicted in Figure 9.

**Figure 10:**
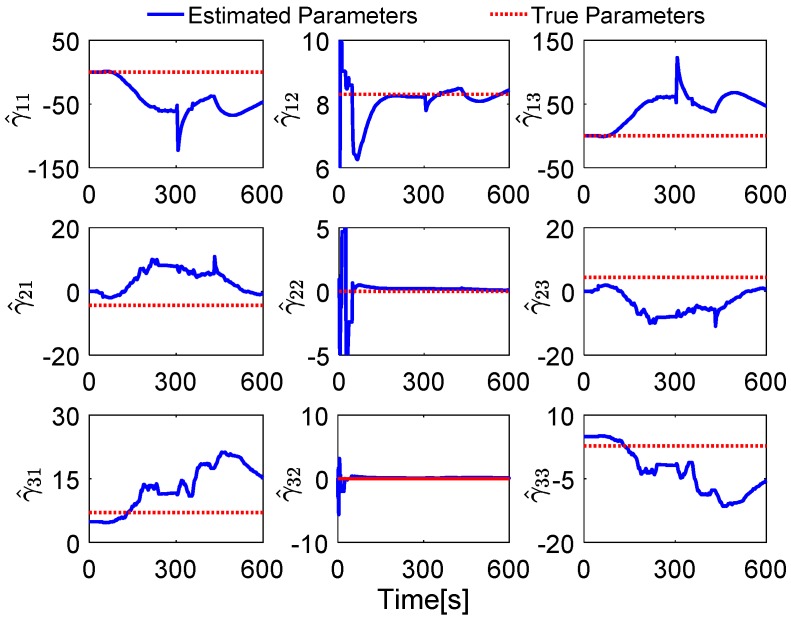
Comparison between estimated parameters and true parameters.

**Figure 11:**
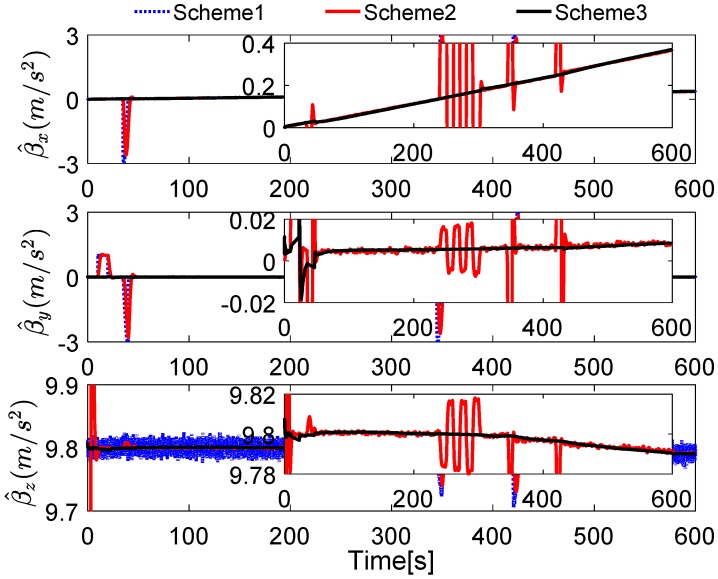
Curves of the reconstructed observation vectors.

**Figure 12:**
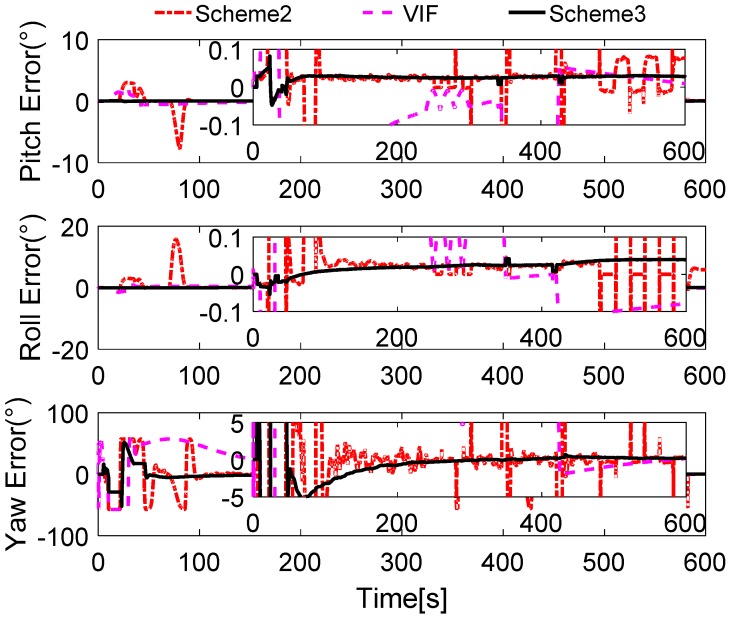
Curves of alignment errors.

To verify the proposed method effectively, the common in-motion states of the vehicle, such as acceleration, deceleration, uniform motion, and turning motion, are considered. In this simulation, the velocity of the vehicle is lower than 10 m/s, and the rotating speed is 6°/s, which are the relative proper in-motion cases for initial alignment. Under the cases of this movement, the outputs of the inertial sensors can be acquired, and the alignment results are shown in Figure 10, Figure 11 and Figure 12. In Figure 10, the curves of vehicle motion is depicted, they are attitude, velocity, and the well-defined trajectory. It is obvious that the moving velocity of the simulation test is not higher than 10 m/s, because the much higher velocity will contaminate the performance of this coarse alignment.

Due to the well-known initial condition of the vehicle, the true parameters are certain. In Figure 10, the estimated parameter matrix is depicted, and the familiar features can be found in Figure 6. As shown, the second parameter of each row of $\hat{\gamma }$ is close to the true parameter, the fluctuation is small, while the other six parameters are wide fluctuations; the reasons have been analyzed in Section 6.1. It is also noted that the parameter ${\hat{\gamma }}_{12}$ is tuning during the whole alignment. This is caused by the real correction of the robust filter, when the new effective measurements are acquired after some outliers, the parameters will be re-estimated. In addition, the other two parameters ${\hat{\gamma }}_{22}$ and ${\hat{\gamma }}_{32}$ are smoother than ${\hat{\gamma }}_{12}$; this is because the gravitational apparent motion is projected on ${\hat{\beta }}_{x}$, and the parameters ${\hat{\gamma }}_{22}$ and ${\hat{\gamma }}_{32}$ are not sensitive to the measurements. The analysis has been confirmed with the reconstructed observation vectors, which is calculated by Equation (32).

In Figure 11, the reconstructed observation vectors of Schemes 1–3 are shown. It can be found that the digital filter cannot eliminate the outliers caused by the vehicle motion, but the robust filter, which is proposed by this paper, can address these defects effectively. It can also be noted that the reconstructed observation vectors ${\hat{\beta }}_{x}$ have significant changes, which consisted of the aforementioned analysis.

In Figure 12, the alignment errors are described. Due to the greater errors in Scheme 1, we do not depict the alignment results of Scheme 1. During the 600 s coarse alignment, the results of Scheme 2 and the VIF method are fluctuating, and they are sensitive to the vehicle motion, thus the results are not suitable for the follow-on inertial navigation. By using the more precisely reconstructed observation vectors, the alignment results of the proposed method are steady and accurate.

For showing the performance of the proposed method, the statistics of the alignment errors during the whole alignment procedure are listed in Table 6.

The statistics of the alignment errors of the proposed method show that the mean value of the final errors of horizontal angles is less than 0.05**°**, and the STD value is under 0.01**°**. When the coarse alignment procedure lasts for 600 s, the mean value of the yaw errors is around 0.2**°**, and the corresponding STD value is under 0.2**°**. All of the errors are low enough for the fine alignment.

### 6.3. Turntable Test

To verify the performance of the proposed method in practical applications, the practical tests, including the turntable test and field vehicle test are designed in this subsection and the next subsection, respectively.

**Figure 13:**
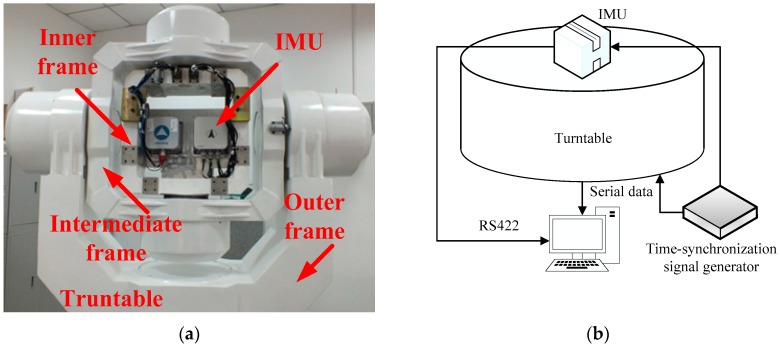
(**a**) Turntable and IMU; and (**b**) construction of turntable test.

For the turntable test, the equipment is installed as shown in Figure 13a, and the construction of the turntable test is as shown in Figure 13b. The turntable is designed by the AVIC Beijing Precision Engineering Institute for the aircraft industry, and the controlling accuracy is ±0.0005°/s, the accuracy of the corresponding angle controlling is ±0.0001°. The IMU used in this test is a navigational-grade production, the corresponding parameters are listed in Table 7.

In this test, the data from turntable and SINS is collected via serial communication ports as a response to the external time-synchronization signal. Before the test, all of the system errors, such as the coupling coincident scale factors of the inertial sensors, installing errors, and so on, are corrected by the calibration test, so the above-mentioned system errors are ignored.

**Figure 14:**
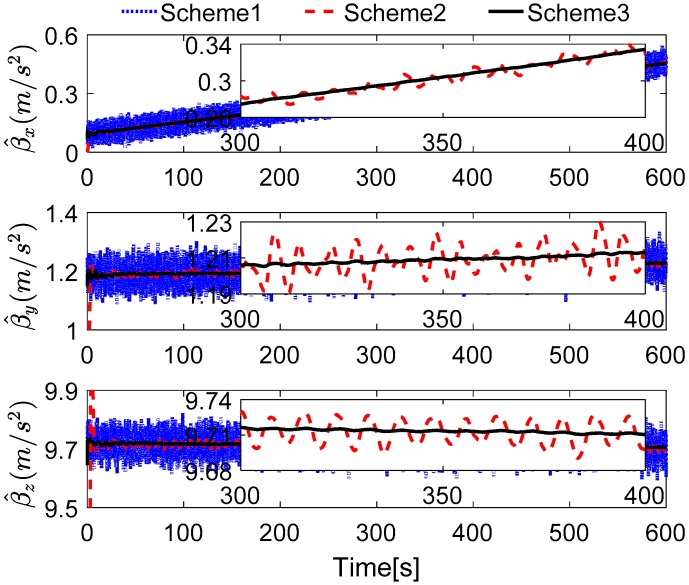
Curves of the reconstructed observation vectors.

**Figure 15:**
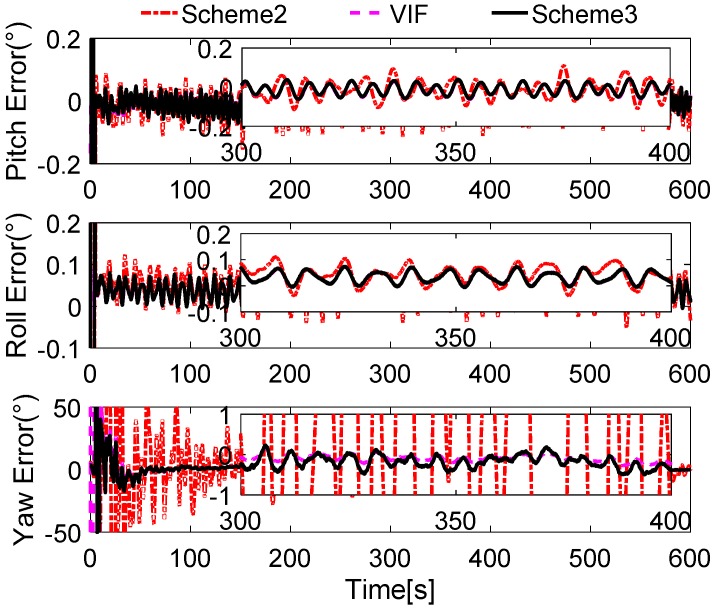
Curves of alignment errors.

The outputs rate of the turntable and SINS are set as 200 Hz, and the turntable works under the swaying condition, the swaying parameters are as common as Table 2. The Kalman filter parameters are set, as shown in Section 6.1, and the robust filter parameter in this test is set as $c_{i}=0.2$, because the magnitude of the noises are greater than the simulation tests. The coarse alignment also lasts for 600 s, the reconstructed observation vectors and the alignment errors are depicted in Figure 14 and Figure 15, respectively.

In Figure 14, it can be found that the reconstructed observation vectors based on Scheme 2 are widely fluctuating, which is caused by the drawbacks of the low-pass digital filter. It is obvious that the reconstructed observation vector, which is acquired by Scheme 3, is smoother than Scheme 2. In addition, it is easily concluded that the smoother observation vector will acquire more stable alignment results, so the alignment results of Scheme 3 will be smoother than Scheme 2.

In Figure 15, the results of Scheme 1 are ignored due to the great interference of the reconstructed observation vectors in Figure 14. The alignment errors are showed that the performance of Scheme 3 is superior to Scheme 2, because the reconstructed observation vectors of Scheme 2 are more widely fluctuating than that which are reconstructed by Scheme 3. Just like in Section 6.1, the partially enlarged views of the alignment errors between 300 s and 400 s are depicted in Figure 15, it can be found that the performance of Scheme 2 is inferior to Scheme 3 and the VIF method, and the horizontal performance of Scheme 3 are familiar with the VIF method. For clear analysis, the statistics of the alignment errors of the three methods from 300 s and 400 s are listed in Table 8.

In Table 8, the mean value of pitch and roll errors of three methods are approximated. However, the STD values of VIF and Scheme 3 are around 0.02**°**, it is smaller than Scheme 2, which is larger than 0.03**°**. Due to the smoothed feature of reconstructed observation vector of Scheme 3, it can be found that the STD value of the yaw error of Scheme 3 is 0.1543**°**, while it is 5.2979**°** of Scheme 2. These features reveal that the alignment results of Scheme 2 are unstable, thus the digital filter cannot obtain the excellent results. Based on the apparent velocity properties, the STD value of the VIF method is 0.1222**°**, and the corresponding value of Scheme 3 is 0.1543**°**. Moreover, the mean value of yaw error of Scheme 3 is −0.1696°, which is −0.1424° for the VIF. It is revealed that the performance of VIF and Scheme 3 is quite equivalent. However, when the alignment is processing under the in-motion case without additional information, Scheme 3 will be superior to the VIF method, and this test is investigated in the next subsection.

### 6.4. Vehicle Test

**Figure 16:**
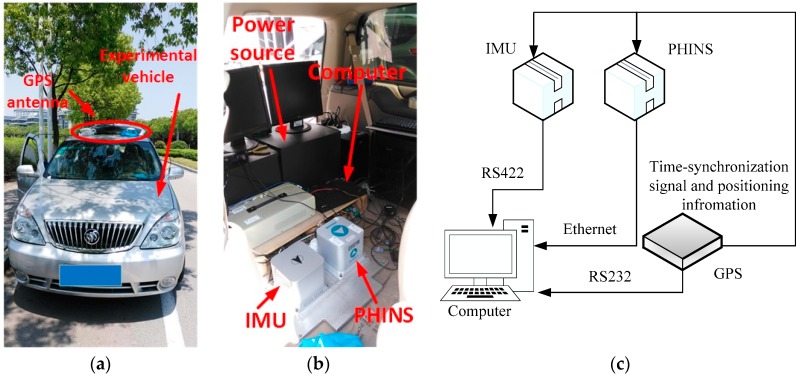
(**a**) Experimental vehicle; (**b**) SINS and PHINS; (**c**) construction of the vehicle test.

In this subsection, the field vehicle test of coarse alignment is designed for examining the performance of Scheme 3. PHINS III, which is produced by iXBlue Corporation (Saint-Germain en Laye Cedex, France), is utilized as the reference system. The experimental vehicle, installed IMU and PHINS and construction of vehicle test are shown in Figure 16a–c, respectively. In Figure 16a, a GPS antenna is used to collect the GPS signal, which is required for PHINS, and then the initial position of the vehicle is well-known. In Figure 16b, the IMU and PHINS are installed on the surface of a steel plate, and the power is supplied with a rechargeable battery pack. All of the raw data of the sensors are logged by the computer. Moreover, a real-time operation system (VxWorks) is embedded in the navigation computer. Four methods mentioned in this paper are processed by four real-time tasks of VxWorks. The alignment results are also logged by the computer. Figure 16c gives the construction of the vehicle test, and the outputs of GPS provide the time-synchronization signal for IMU and PHINS. The positioning information of GPS is also acquired by PHINS and computers via serial communication ports. The PHINS data are collected via Ethernet, and the raw data of the outputs of the inertial sensors are transferred via an RS422 port.

**Figure 17:**
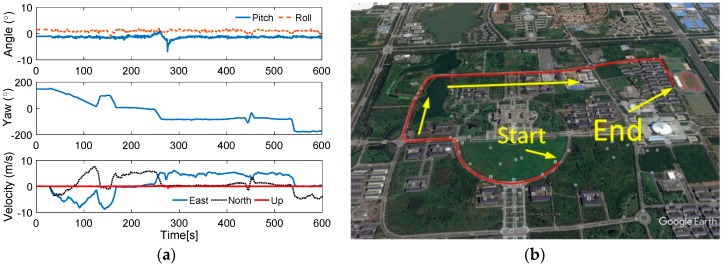
(**a**) Curves of vehicle motion; and (**b**) trajectory of vehicle motion.

Before the test, the installed error between IMU and PHINS are corrected, and the IMU system errors, such as the coupling coincident scale factors of the inertial sensors, are also corrected by the calibration methods. In Figure 17, the curves and trajectory of vehicle motion are depicted, and the field test is proceeded on our campus. The velocity of the vehicle is under 10 m/s.

**Figure 18:**
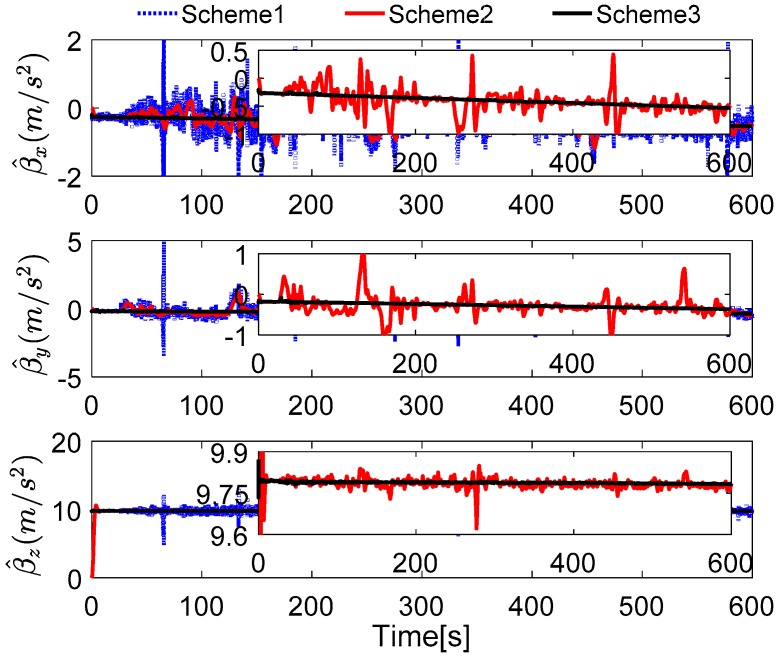
Curves of the reconstructed observation vectors.

**Figure 19:**
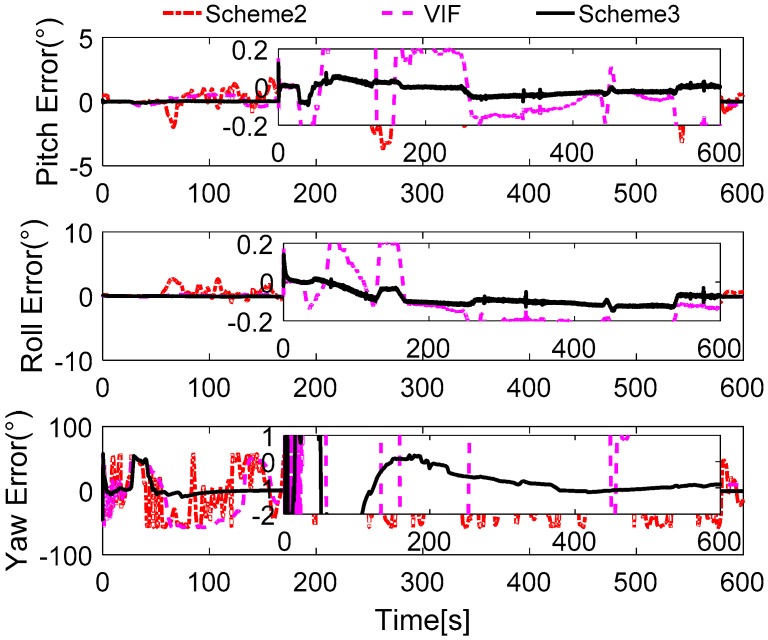
Curves of alignment errors.

The field test designed here lasts for 600 s, and the reconstructed observation vectors and the alignment errors are shown in Figure 18 and Figure 19, respectively. In Figure 18, the reconstructed observation vectors of the three methods are shown, and the wide fluctuation in Scheme 1 can be found, which is because the disturbance of the vehicle movement, the external environment, and the sensors’ noises. Based on the IIR low-pass digital filter, some high-frequency noises are filtered out from the observation vectors. The enlarged views of the reconstructed observation vectors are shown that there are a lot of outliers in Scheme 2, these outliers will contaminate the performance of the coarse alignment. Based on Scheme 3, the outliers are address by the robust filter, and the optimal observation vectors are extracted by the parameter recognition and the reconstruction algorithm, which shows that the reconstructed observation vectors of Scheme 3 are more stable. The alignment errors showed in Figure 19 also verify the precision of the extracted observation vectors of Scheme 3.

In Figure 19, it is obviously found that the alignment results of Scheme 2 are wide fluctuation and it does not acquired a stable value after 600 s. In this test, the SINS under an entirely self-contained mode is considered. Hence, the performance of the VIF method is also poor in this field test, because the additional information, which is the external reference velocity, cannot be acquired when SINS is under an entirely self-contained mode. According to the aforementioned analysis, the reconstructed observation vectors of Scheme 3 have been reconstructed by the designed method, and the precision of these vectors can be verified by the alignment errors. It is noted that the performance of Scheme 3 is superior to the other two methods, and the errors of pitch and roll are less than 0.2° during the whole alignment procedure. The errors of yaw are constraint in 2°, when the errors are stable. It is also can be found that the smaller distortion of the alignment errors of Scheme 3, and this is caused by the interference of the vehicle movements, which disturb the measurement observation vectors. In order to show the precision of Scheme 3, Table 9 summarized the statistics of the alignment errors of Scheme 3.

In Table 9, it is shown that the STD value of the errors of pitch and roll are less than 0.1°, and the corresponding value of yaw errors is less than 0.5° after the alignment lasts for 100 s. These reveal that the alignment results of Scheme 3 are available in the practical system.

## 7. Conclusions

In this work, a coarse alignment method based on apparent gravity are proposed, and the mechanisms of inertial frame alignment are studied at first. Then, an IIR low-pass digital filter is utilized to filter the high-frequency noises, which are contained in the measurement observation vectors. Thirdly, to extract the apparent gravitational motion precisely from the observation vectors, a parameter recognition and vector reconstructed method are designed. Alternatively, a robust filter is investigated to address the interference of the gross outliers, which is caused by the varying velocity movement. Finally, the simulation and physical tests are designed for verifying the performance of the proposed method. The results of the comprehensive tests show that the performance of the proposed method is equivalent to the current popular method on a sawing base, which is a VIF method. However, it is superior to the VIF method on the moving base. Based on the numerical analysis, we can conclude that the proposed method is available for practical systems, and it also can be designed as a new gyrocompassing method in future work.

## Figures and Tables

**Table 1:** The parameters of digital filter.

Digital Filter Parameters			
Apass (dB)	3	Orders	Minimum order
Astop (dB)	80	Structure	Direct-form II
Fpass (Hz)	0.1	Fs (Hz)	200
Fstop (Hz)	1	Designed method	Butterworth

**Table 2:** The swaying parameters.

Items	Pitch	Roll	Yaw
Amplitude (°)	10	12	6
Frequency (Hz)	0.2	0.125	0.15
Initial phase (°)	0	0	0
Swaying center (°)	0	0	0

**Table 3:** Sensor errors.

Axes	Gyro Noise (°/h)		Accelerometer Noise (μg)	
	Constant	Random	Constant	Random
*x*-axis	0.01	0.01	500	500
*y*-axis	0.01	0.01	500	500
*z*-axis	0.01	0.01	500	500

**Table 4:** The statistics of the alignment errors from 300 s to 400 s (°).

Methods	Pitch		Roll		Yaw	
	Mean	STD	Mean	STD	Mean	STD
Scheme 2	0.0280	0.0041	−0.0198	0.0062	0.0221	0.5366
Scheme 3	0.0280	0.0019	−0.0257	0.0027	−0.0307	0.0904
VIF	0.0280	0.0020	−0.0269	0.0021	0.0402	0.0098

**Table 5:** The process of the vehicle motion.

Time (s)	State
0–10	Stationary State
10–20	Accelerated motion(a = 1 m/s^2^)
20–30	Uniform motion(v = 10 m/s)
30–45	Turn left motion(w = 6°/s)
45–240	Uniform motion(v = 10 m/s)
240–300	Accelerated and decelerated motion (three times)
300–340	Uniform motion(v = 10 m/s)
340–355	Turn left motion(w = 6°/s)
355–415	Uniform motion(v = 10 m/s)
415–430	Turn left motion(w = 6°/s)
430–600	Uniform motion(v = 10 m/s)

**Table 6:** The statistics of the alignment errors (°).

Time(s)			1–100	101–200	201–300	301–400	401–500	501–600
Scheme 3	Pitch	Mean	0.0176	0.0278	0.0248	0.0248	0.0272	0.0293
		STD	0.0229	0.0013	0.0006	0.0042	0.0038	0.0047
	Roll	Mean	−0.0099	0.0148	0.0210	0.0253	0.0304	0.0392
		STD	0.0145	0.0028	0.0019	0.0038	0.0070	0.0064
	Yaw	Mean	0.2445	−1.6114	−0.3178	0.0065	0.3011	0.2005
		STD	18.3219	0.7545	0.1522	0.1637	0.1271	0.1303

**Table 7:** Sensor parameters.

**Gyroscope**			
Constant bias	<0.02°/h(1σ)	Nonlinearity of scale factor	≤50 ppm(1σ)
Repetitiveness of constant bias	<0.01°/h(1σ)	Repetitiveness of scale factor	≤50 ppm(1σ)
Random walk	$<0.005{}^{\circ}/\sqrt{h}$	Measuring range	−300~+300°/s
**Accelerometer**			
Measuring range	−20~+20 g	bias	$<5\times 10^{-4}\;g$
Threshold	$<5\times 10^{-6}\;g$	Temperature coefficient of bias	$<6\times 10^{-5}/℃$
			(−40~+40 ℃)
Repetitiveness of scale factor	$<3.5\times 10^{-5}\;g\left(1\sigma \right)$	Repetitiveness of bias	$<2.5\times 10^{-4}\;g\left(1\sigma \right)$
Temperature coefficient of scale factor	$<6\times 10^{-5}/℃$	bandwidth	>800 Hz
	(−40~+40 ℃)		

**Table 8:** The statistics of the alignment errors from 300 s to 400 s (°).

Methods	Pitch		Roll		Yaw	
	Mean	STD	Mean	STD	Mean	STD
Scheme 2	−0.0107	0.0460	0.0004	0.0334	−0.3676	5.2979
Scheme 3	−0.0098	0.0274	−0.0101	0.0211	−0.1696	0.1543
VIF	−0.0115	0.0276	−0.0095	0.0210	−0.1424	0.1222

**Table 9:** The statistics of the alignment errors (°).

Time (s)			1–100	101–200	201–300	301–400	401–500	501–600
Scheme 3	Pitch	Mean	0.0060	0.0164	−0.0243	−0.0417	−0.0252	−0.0076
		STD	0.0413	0.0129	0.0236	0.0055	0.0058	0.0142
	Roll	Mean	−0.0028	−0.0730	−0.1033	−0.1017	−0.1198	−0.0908
		STD	0.0250	0.0260	0.0097	0.0054	0.0084	0.0256
	Yaw	Mean	5.6825	−0.4104	−0.4767	−0.9022	−1.1007	−0.9433
		STD	18.3266	0.7514	0.2104	0.1263	0.0379	0.0460

**Figure A1:**
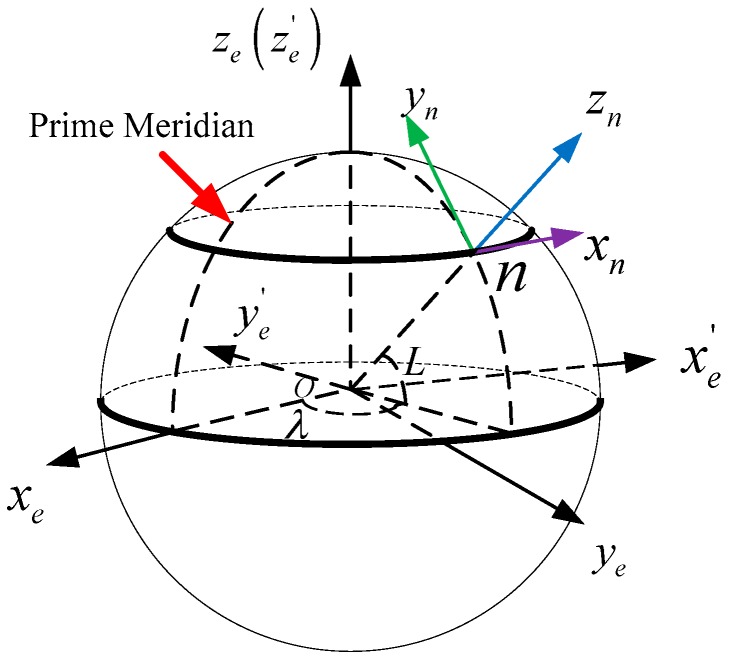
Curves of alignment errors.

## Abbreviations

The following abbreviations are used in this manuscript:

SINS	Strapdown Inertial Navigation System
ENU	East–North–Up
IMU	Inertial Measurement Unit
ECEF	Earth-Centered Earth-Fixed
DCM	Direction Cosine Matrix
STD	Standard Deviation
GPS	Global Positioning System

## Appendix A

The derivation of the parameter matrix in Equation (18) is shown in this appendix. According to Equation (17), the DCM $C_{e0}^{b0}$ can be split into by the chain rules of DCM:

(A1) $$C_{e0}^{b0}=C_{n0}^{b0}C_{e0}^{n0}$$

where $C_{n0}^{b0}$ is the DCM, which is related to the attitude of SINS at start up. Due to the shorter time of the coarse alignment, the DCM $C_{e(t)}^{n(t)}$ can be approximated to ${\left(C_{e0}^{n0}\right)}^{T}$. Since the rotation rate $\omega_{ie}$ of the Earth is well-known, the DCM $C_{e\left(t\right)}^{e0}$ can be calculated by

(A2) $$C_{e\left(t\right)}^{e0}=\left[\begin{array}{ccc} \cos\left(\omega_{ie}t\right) & -\sin\left(\omega_{ie}t\right) & 0\\ \sin\left(\omega_{ie}t\right) & \cos\left(\omega_{ie}t\right) & 0\\ 0 & 0 & 1 \end{array}\right].$$

Therefore, it is focused on calculating the DCM $C_{n\left(t\right)}^{e\left(t\right)}$. In Figure A1, we depict the transfer process from the *e*-frame to the *n*-frame.

It is shown that it needs two step transformations from the *e*-frame to the *n*-frame, the first step is to turn the *e*-frame 90+ λ degrees around the $z_{e}$-axis, so that it can be obtain the *e’*-frame, and then to turn the *e’*-frame 90− L degrees around $x_{e\prime }$-axis. Thus, the procedure can be described as

(A3) $$C_{n\left(t\right)}^{e\left(t\right)}=C_{e^{\prime }\left(t\right)}^{e\left(t\right)}C_{n\left(t\right)}^{e^{\prime }\left(t\right)}=\left[\begin{array}{ccc} \cos\left(90+\;\lambda \right) & -\sin\left(90+\;\lambda \right) & 0\\ \sin\left(90+\;\lambda \right) & \cos\left(90+\;\lambda \right) & 0\\ 0 & 0 & 1 \end{array}\right]\left[\begin{array}{ccc} 1 & 0 & 0\\ 0 & \cos\left(90-\;L\right) & -\sin\left(90-\;L\right)\\ 0 & \sin\left(90-\;L\right) & \cos\left(90-\;L\right) \end{array}\right].$$

After some manipulations, the matrix $-C_{e0}^{n0}C_{e\left(t\right)}^{e0}C_{n\left(t\right)}^{e\left(t\right)}g^{n}$ can be described as

(A4) $$-C_{e0}^{n0}C_{e\left(t\right)}^{e0}C_{n\left(t\right)}^{e\left(t\right)}g^{n}=\left[\begin{array}{ccc} 0 & g\cos\left(L\right) & 0\\ -g\sin\left(L\right)\cos\left(L\right) & 0 & g\sin\left(L\right)\cos\left(L\right)\\ g\cos^{2}\left(L\right) & 0 & g\sin^{2}\left(L\right) \end{array}\right]\left[\begin{array}{c} \cos\left(\omega_{ie}t\right)\\ \sin\left(\omega_{ie}t\right)\\ 1 \end{array}\right].$$

When the initial position is well-known, the above matrix will be calculated precisely.

## Appendix B

The derivation of robust filter is shown in this appendix. It is assumed that a common a linear time-invariant model can be expressed as

(A5) $$\{\begin{array}{c} x_{k+1}=\phi x_{k}+w_{k}\\ z_{k+1}=Hx_{k+1}+v_{k+1} \end{array}$$

where $w_{k}$ and $v_{k+1}$ are white Gaussian noises, and

(A6) $$\{\begin{array}{c} E\left[w_{k}\right]=0,\;E\left[w_{k}{\left(w_{k}\right)}^{T}\right]=Q_{k}\\ E\left[v_{k+1}\right]=0,\;E\left[v_{k+1}{\left(v_{k+1}\right)}^{T}\right]=R_{k+1} \end{array}.$$

Then, the general weight least-square criterion can be given by

(A7) $$J={\left(z-Hx\right)}^{T}R^{-1}\left(z-Hx\right)+{\left(x-\bar{x}\right)}^{T}P^{-1}\left(x-\bar{x}\right)$$

where $\bar{x}$ is one-step prediction of x, and P can be calculated by

(A8) $$P=E\left[\left(x-\bar{x}\right){\left(x-\bar{x}\right)}^{T}\right].$$

According to gradients of real valued functions with vector arguments, that is, if x(m×1), y(n×1), A(n×m), and B(n×n), then

(A9) $$\frac{\partial \left({\left(y-Ax\right)}^{T}B\left(y-Ax\right)\right)}{\partial x}=-2A^{T}B\left(y-Ax\right).$$

Consequently, the optimization function J can be minimized by

(A10) $${\frac{\partial J}{\partial x}|}_{x={\hat{x}}_{k+1}}=-2H^{T}R_{k+1}^{-1}\left(z_{k+1}-H{\hat{x}}_{k+1}\right)-2P_{k,k+1}^{-1}\left({\hat{x}}_{k+1,k}-{\hat{x}}_{k+1}\right)=0.$$

Additionally, the following can be easily acquired:

(A11) $${\hat{x}}_{k+1}={\hat{x}}_{k+1,k}+{\left(H^{T}R_{k+1}^{-1}H+P_{k,k+1}^{-1}\right)}^{-1}H^{T}R_{k+1}^{-1}\left(z_{k+1}-H{\hat{x}}_{k+1,k}\right).$$

According to Equation (28), the weight least-square criterion in Equation (A7) be modified as

(A12) $$J_{i,k+1}^{\prime }={\left({\bar{\beta }}_{i,k+1}-\gamma_{i,k}M_{k+1}\right)}^{2}S_{i,i}+{\left(\gamma_{i,k}-{\hat{\gamma }}_{i,k}\right)}^{T}P_{i,k}^{-1}\left(\gamma_{i,k}-{\hat{\gamma }}_{i,k}\right).$$

By minimizing the criterion function in Equation (A12), the following can be obtained:

(A13) $$\{\begin{array}{c} {\hat{\gamma }}_{i,k+1}={\hat{\gamma }}_{i,k},\;\mathrm{if}\;{\left|{\bar{\beta }}_{k+1}-{\hat{\gamma }}_{k}M_{k+1}\right|}_{i}^{2}>c_{i}\\ {\hat{\gamma }}_{i,k+1}={\hat{\gamma }}_{i,k}+{\left(\frac{M_{k+1}M_{k+1}^{T}}{\Lambda_{i,k+1}}+P_{i,k}^{-1}\right)}^{-1}\frac{M_{k+1}}{\Lambda_{i,k+1}}\left({\bar{\beta }}_{i,k+1}-{\hat{\gamma }}_{i,k}M_{k+1}\right),\;\mathrm{otherwise} \end{array}$$

where the subscript i denotes the ith row of the parameter matrix γ. Consequently, the robust filter can be rearranged as Equations (30)–(33).
